# Challenges in diagnosis and management of perineal hypospadias with a testicular seminoma mimicking paratesticular tumor in a solitary testis: A case report

**DOI:** 10.1016/j.eucr.2022.102050

**Published:** 2022-03-16

**Authors:** Ramlan Nasution, Ali Husein, Lidya Imelda Laksmi, Belman Novenry Silalahi

**Affiliations:** a Division of Urology, Department of Surgery, Faculty of Medicine, Haji Adam Malik Hospital, Universitas Sumatera Utara, Indonesia; b Department of Anatomical Pathology, Faculty of Medicine, Universitas Sumatera Utara, Indonesia

**Keywords:** Hypospadias, Seminoma

## Abstract

Diagnosing and managing perineal hypospadias in patient with a suspected paratesticular seminoma in a solitary testis may be challenging. We describe a case of 26-year-old male presented with painless left scrotal swelling with hypospadias and chordee. The patient underwent two-staged surgery: chordectomy and open inguinal testicular biopsy followed by urethroplasty and radical orchidectomy.

## 1 Introduction

Diagnosing and managing perineal hypospadias in patient with a suspected paratesticular seminoma in a solitary testis may be challenging since the clinical presentation might come late and a quick decision is needed. In the presence of the paratesticular seminoma, we should always suspect of seminoma foci in the testicle.^1^ On the other side, hypospadias is the most common congenital abnormality of the urethra. The fact that there are various surgical options and techniques for hypospadias correction shows that no surgical approach guarantees universal success.^2^

The paper presents case report of a young man with perineal hypospadias and left testicular seminoma. This present case challenged the team, especially the condition of the patient who had a solitary testis and unmarried which required an interdisciplinary approach.

## 2 Case presentation

**Fig. 1:**
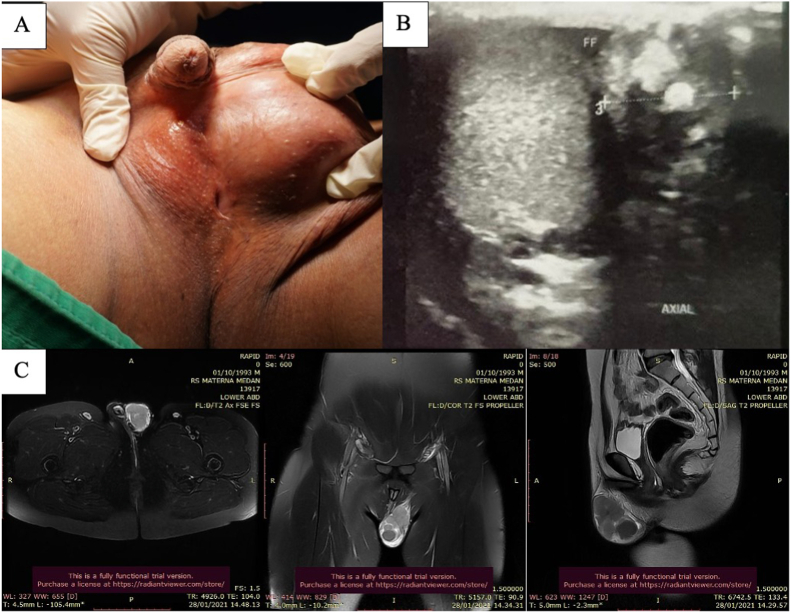
Preoperative evaluation A. Clinical Picture of the Left Paratesticular Tumor and Scrotal Hypospadia B. Perioperative Testicular USG C. MRI of patient with paratesticular seminoma.

A 26-year-old male came to our hospital with chief complaint of left scrotal swelling which arises 3 months ago. The mass was painless and grew in size. We also found that the patient did not have any right testicle since birth. The physical examination on the external genitalia also revealed a perineal hypospadias with chordee. No scars were found during the testicle examination (Fig. 1). The patients never seek any medical treatment due to economic issue. All of the laboratory parameter for testicular tumour were in the normal range (LDH: 427 U/L; alfa-fetoprotein: 1,5 ng/mL; and β-hCG: 2,0 mIU/mL). Based on the MRI evaluation (Fig. 1), we found a septated mass sized 5cmx3cm in left paratesticular. On the contralateral, no testicular body was found.

**Fig. 2:**
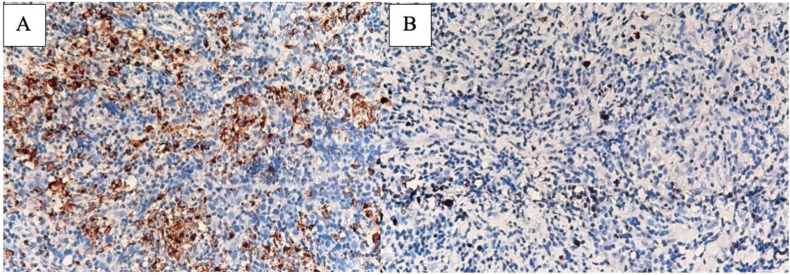
CD117 and KI67 IHC staining A. 400x. High power magnification showing the positivity with CD117 (c-KIT). B. 400x. High power magnification showing the positivity with Ki67.

On January 2021, we performed chordectomy and *trans*-inguinal exploration on the left scrotal mass. In addition, tumor biopsy was performed to identify the tumour nature. The histopathological result led to a malignant lesion of round blue cell tumour with high suspicion of Non-Hodgkin Lymphoma. However, due to inconclusive characteristic the specimen was then sent to underwent immunohistochemistry (IHC) staining. Immunohistochemistry showed positive result for CD68, CD117 (c-Kit), and Ki67 thus consistent with seminoma (Fig. 2).

**Fig. 3:**
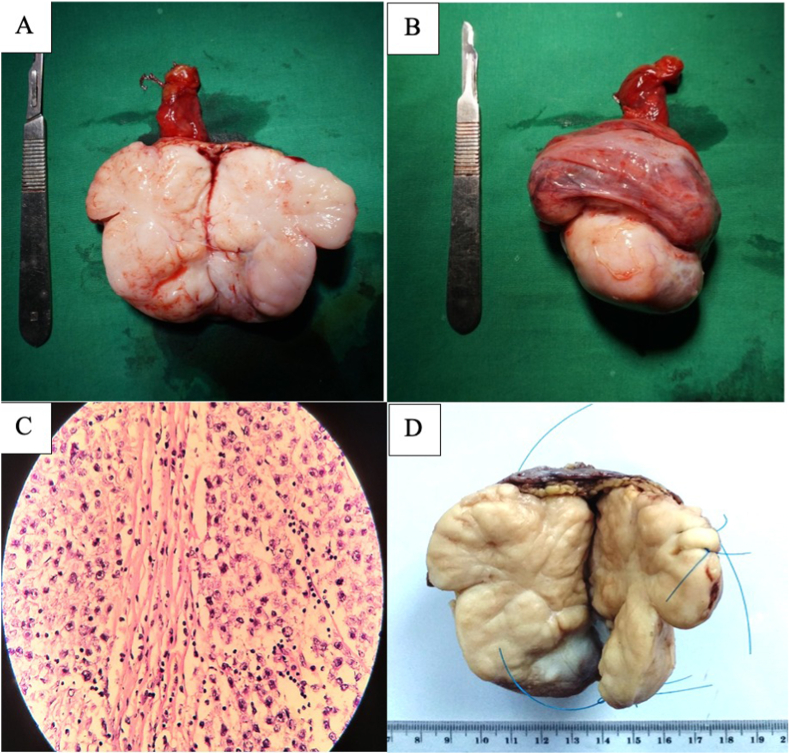
Postoperative evaluation A. Tumo r resected from epydidimis (1) B. Tumo r resected from epydidimis (2) C. Microscopic appearance. The tumour cells have polygonal nuclei with centrally located nucleoli. The cytoplasm was pale and clear. The tumour was interrupted by fibrovascular septa containing lymphocytes. (HE, 400x) D. Macroscopic appearance. The mass was grayish and multinodular.

On the post-surgical physical examination, the mass grew in size from 5cm to 8cm and there was also a stone-hard nodul in the left testis. The second operation was planned to perform left radical orchidectomy and urethroplasty. The patient offered for sperm preservation but he declined and consented with the surgical procedure. Then, radical orchidectomy and urethroplasty was performed (Fig. 3). Grossly, the testis was soft and grayish. The mass was solid and the size was 7cm × 5cm × 3cm. The size of spermatic cord was 4,5cmx1,5cmx1cm. The mass on testis and spermatic cord showed the same microscopic appearance. They consisted of diffuse pleomorphic cells, interrupted by fibrovascular septa. The nuclei was polygonal with finely granular chromatin and centrally located nucleoli. The tumour cells invaded lymphovascular and also found in epididymis and tunica albugenia (Fig. 3). Post surgery serum tumour markers evaluation were within the normal limit. Therefore this case is concluded as a testicular seminoma pT_2_N_0_M_0_S_0_.

## 3 Discussion

Paratesticular seminoma usually associated with seminoma lesion of the testicle. It is extremely rare case and we should suspect of seminoma foci in the testicle if we found the presence of paratesticular seminoma. Case report of paratesticular seminoma described by Dutkiewicz et al. reported there were micro-foci of seminoma in the testis.^1^ The literature has accounts of epididymal seminoma, but upon closer examination, these were all associated with foci of seminoma within the testis and were thought to arise from the testis rather than from the epididymis itself, and they were all regarded to arise from the testis. A thorough search of the available literature revealed that there had been just one previous recorded case of a true primary epididymal seminoma,^3^ prior to this study.

After the first surgery our team provides counselling to the patient and family for sperm preservation considering the patient's unmarried status. Related to the infertility issue, a number of researchers have recommended cryopreservation of sperm before treatment for TGCT, as infertility can harm young patient's mental health.^4^ However, after the discussion with the family, the patient decided to refuse the sperm analysis examination. The histopathological result and post surgery serum tumor markers evaluation revealed a testicular seminoma pT_2_N_0_M_0_S_0_. Furthermore, according to the NCCN 2021,^5^ the patient is categorized at stage IB. The NCCN Panel prefers surveillance to adjuvant therapy.

## 4 Conclusion

To the best of our knowledge, this is the first reported case of testicular seminoma with hypospadias in a solitary testis. Effective management of multiple diseases requires a multimodal, interdisciplinary approach.
